# Ultra-broadband on-chip beam focusing enabled by GRIN metalens on silicon-on-insulator platform

**DOI:** 10.1515/nanoph-2022-0242

**Published:** 2022-07-14

**Authors:** Jian Shen, Yong Zhang, Yihang Dong, Zihan Xu, Jian Xu, Xueling Quan, Xihua Zou, Yikai Su

**Affiliations:** State Key Lab of Advanced Optical Communication Systems and Networks, Department of Electronic Engineering, Shanghai Jiao Tong University, Shanghai 200240, China; Center for Advanced Electronic Materials and Devices, Shanghai Jiao Tong University, Shanghai 200240, China; Center for Information Photonics and Communications, School of Information Science and Technology, Southwest Jiao Tong University, Chengdu 611756, China

**Keywords:** GRIN metalens, integrated devices, on-chip beam focusing, silicon photonics, subwavelength structures

## Abstract

Metalens has emerged as an important optical block in free-space optical systems, which shows excellent performance. Even the metalens based on gradient index (GRIN) profiles can be implemented for on-chip beam focusing behavior. However, for most previous schemes, the GRIN metalenses can only achieve on-chip beam focusing behavior in one dimension, which limits their applications in low-loss waveguide interconnecting or fiber-to-chip coupling. In this paper, an on-chip half Maxwell’s fisheye lens based on GRIN profiles with subwavelength features, integrated with silicon waveguides, is experimentally demonstrated. Benefitting from the index distribution and beam focusing characteristics of the half Maxwell’s fisheye lens, an on-chip beam transforming can be achieved for transverse electric (TE) fundamental mode in two waveguides with different heights and widths. The simulated 1 dB bandwidth can reach 1100 nm, which exhibits great prospects in integrated photonic circuits. The measured insertion loss of an on-chip 5.4 μm-length lens is less than 1 dB to connect a 220 nm-height, 8 μm-wide waveguide, and a 60 nm-height, 0.5 μm-wide waveguide in the wavelength range of 1280–1620 nm.

## 1 Introduction

Silicon platform that can offer high index contrast and low waveguide loss is considered to be promising in large-scale integrated photonic circuits [1–5]. Thanks to the complementary-metal-oxide-semiconductor (CMOS)-compatible process, many on-chip silicon photonic devices have been implemented at low cost [6–10]. Adiabatic tapers are essential components to achieve the beam transforming between waveguides with different sizes in the integrated photonic circuits, such as segmented taper [11], sinusoidal taper [12], and hollow taper [13]. Fiber-to-chip coupler can realize the beam transforming between two waveguides with different heights and widths. Many fiber-to-chip couplers have been proposed based on multi-tip tapers structures [14–16], multilayer waveguides structures [17, 18], and lens-assisted structures [19–22]. However, these structures may face the challenges of a large footprint and limited operation bandwidth. Recently, an 850-μm-long adiabatic taper was demonstrated with beam transforming behavior in two waveguides with different heights and widths, which found application for fiber-to-chip coupling [23]. A wedge-like structure defined by the SU-8 film was constructed as an adiabatic tapered waveguide. However, the length of the tapered waveguide is long. On-chip GRIN profiles have been implemented on silicon-on-insulator (SOI) substrates to realize beam focusing with a compact footprint [24–26]. Metasurface [27–29] and subwavelength grating [30–32] are employed to achieve the desired on-chip GRIN profiles. Beam transforming between waveguides with different widths has been implemented based on an on-chip Luneburg lens [26]. However, only one-dimensional beam transforming is realized by previous reported GRIN-based spot size converters.

Compared to conventional 220 nm-height silicon waveguides, the ultrathin silicon waveguides exhibit unique characteristics of lower propagation loss, strong evanescent field, and less nonlinear effect [33]. Many applications based on the ultrathin silicon waveguides have been implemented, such as on-chip optical delay lines [34], integrated high-speed modulators [35, 36], evanescent field sensing [37], and fiber-to-chip edge coupler [38]. Therefore, it is highly desired to realize a compact device with a beam transforming in two waveguides with different heights and widths, especially in the height dimension.

In this paper, we experimentally demonstrate an integrated half Maxwell’s fisheye lens based on GRIN profiles, which is offered by engineering the filling ratios of the periodic silicon nanorods with subwavelength features on an SOI substrate. Benefitting from the on-chip index distribution and focusing characteristics of the 5.4 μm-length lens, a beam transforming for TE fundamental mode is achieved between a 220 nm-height, 8 μm-wide waveguide, and a 60 nm-height, 0.5 μm-wide waveguide. The simulated 1 dB operation bandwidth can reach 1100 nm. Due to the wavelength limitation of our experimental setups, the measured bandwidth is 340 nm with insertion losses of <1 dB. In terms of ultra-broadband operation bandwidth, low insertion loss, and compact footprint, the proposed lens-assisted beam transforming in two waveguides with different heights and widths is promising to find applications for waveguide interconnecting or fiber-to-chip coupling in large-scale integrated photonic circuits.

## 2 Design of the integrated GRIN metalens

**Figure 1:**
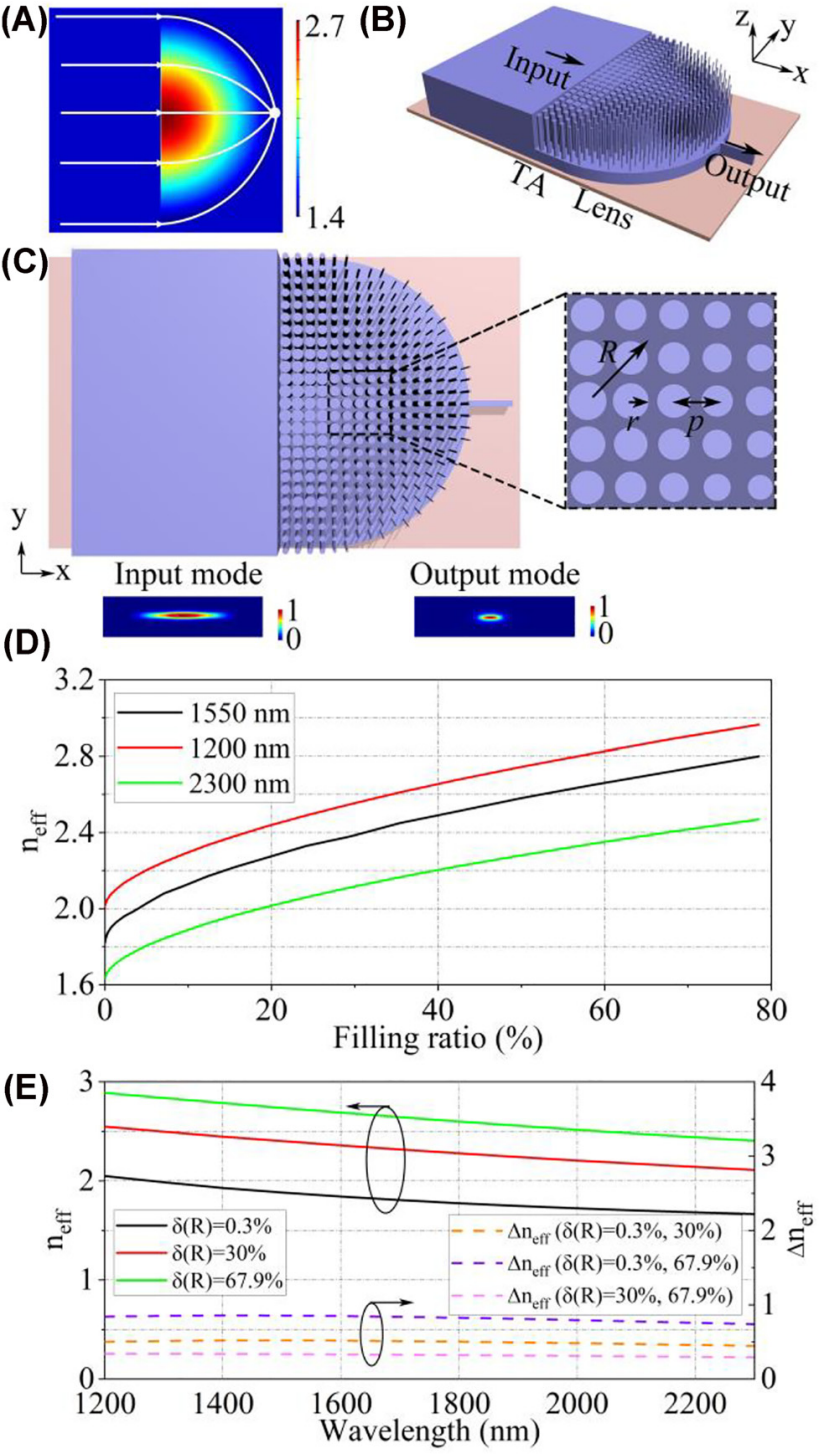
Design of half Maxwell’s fisheye lens. (A) Beam focusing behavior of the lens (B) Schematic of the on-chip half Maxwell’s fisheye lens. (C) *Xy*-plane view of the lens and magnified view of the nanorods array. (D) Relationship between the calculated effective index (*n*_eff_) and the filling ratios (*δ*(*R*)) of nanorods. (E) Relationship between the calculated effective index (*n*_eff_) and the wavelength. TA: transition area.

As illustrated in Figure 1(A), the beam focusing behavior is implemented by a half Maxwell’s fisheye lens. After passing through an ideal half Maxwell’s fisheye lens, the incident parallel lights in different locations are focused to an identical point on the hemispherical circumference [39, 40]. In theory, the refractive index distribution *n*(*R*) and the phase distribution of an ideal half Maxwell’s fisheye lens can be expressed as [27, 41]:

(1) $$n\left(R\right)=\frac{n_{\max}}{1+{\left(R/R_{\max}\right)}^{2}},$$

(2) $$\varphi \left(x,y\right)=\frac{2\pi }{\lambda_{c}}n_{\text{eff}}\left(R\right)\left(f-\sqrt{f^{2}+x^{2}+y^{2}}\right),$$

where *n*_max_, *R*, and *R*_max_ represent the maximum refractive index, radial distance, and radius of the half Maxwell’s fisheye lens, respectively. The effective refractive index *n*_eff_(*R*) of the on-chip GRIN metalens varies along the *x* and *y* direction and *f* is the focal length. It indicates that a refractive index relationship of *n*_max_ = 2*n*_min_ is required in the lens, where *n*_max_ and *n*_min_ are the indices at the origin and edge of the semi-circle of the lens, respectively. The three-dimensional (3D) view of the proposed on-chip GRIN metalens is plotted in Figure 1(B), including the semicircle region of the lens, input and output waveguides, and transition area (TA) with tapered silicon nanorods. In our design, the GRIN profiles required by the half Maxwell’s fisheye lens are mapped on the metamaterial layer that consists of periodic silicon nanorods arrays. The refractive indices of the silicon nanorods are achieved by [42, 43]:

(3) $$n_{\text{meta}}^{2}\left(R\right)=\delta \left(R\right)\cdot n_{\text{Si}}^{2}+\left[1-\delta \left(R\right)\right]\cdot n_{\text{Si}O_{2}}^{2},$$

where *n*_meta_(*R*) denotes the equivalent material index of the on-chip metamaterial layer for polarization parallel (along the *y*-axis direction) to the xy plane, *n*_Si_ and *n*_SiO2_ indicate the inherent material indices of silicon and silica. The GRIN metalens exhibits anisotropic characteristics, but this anisotropy will not affect the focusing property (see Section 1 of the Supplementary Information) [26]. As shown in Figure 1(C), the filling ratio *δ*(*R*) can be described as *πr* ^*2*^/*p* ^*2*^ at the radial distance *R*, where *r* is the radius of the silicon nanorods and *p* is the period of the silicon nanorods array. For TE fundamental mode, the effective refractive index *n*_eff_(*R*) in the metamaterial layer with 60 nm-height silicon slab and 160 nm-height silicon nanorods can be calculated by the finite element method. Then, the relationship between the effective refractive indices *n*_eff_ *(R)* and the equivalent material indices *n*_meta_(*R*) can be obtained. The effective refractive index is calculated to increase from 1.82 to 2.8 with the equivalent material index ranging from 1.44 to 3.47. Therefore, according to the look-up table of the *n*_meta_ *(R)* and *δ(R)* following the law of Eq. (3), the relationship between *n*_eff_(*R*) and *δ*(*R*) can be achieved, as plotted in Figure 1(D). The effective refractive index *n*_eff_(*R*) is dependent on the wavelength. Theoretically, the phase of light after passing through the waveguide can be expressed as [44]:

(4) $$\phi =\phi_{\text{0}}+n_{\text{eff}}k_{0}L,$$

where *φ*_0_ is the initial phase of the light, *n*_eff_ is the effective refractive index of the waveguide, and *k*_0_ = 2π/λ is the wave number. For only one wavelength, the phase difference caused by different widths of the waveguides can be achieved by:

(5) $$\Delta \phi =\frac{2\pi }{\lambda }\left[n_{\text{eff}}\left(h_{1},\omega_{1}\right)-n_{\text{eff}}\left(h_{2},\omega_{2}\right)\right]\cdot L,$$

where *n*_eff_ *(h*_1_ *, ω*_1_ *)* and *n*_eff_ *(h*_2_ *, ω*_2_ *)* are the effective refractive index of the waveguides with different widths of *ω*_1_ *, ω*_2_ and different heights of *h*_1_ *, h*_2_. According to Eq. (5), for only one wavelength, the phase difference can be controlled by adjusting the width and height of the waveguide. However, when multiple wavelengths are introduced, the relationship between wavelength and effective refractive index must be considered. Similarly, this is the main cause of the chromatic aberration presented in the on-chip lens. Therefore, a good solution to this problem will increase the operation bandwidth accordingly.

The phase distribution of the on-chip GRIN metalens has been given in Eq. (2). Therefore, a chromatic aberration-free GRIN metalens satisfies the following conditions:

(6) $$\begin{array}{ll} \Delta \varphi_{a,b}\left(x,y\right) & =\frac{2\pi }{\lambda_{a,b}}\left[n_{\text{eff}}\left(\delta_{a},\lambda_{a,b}\right)-n_{\text{eff}}\left(\delta_{b},\lambda_{a,b}\right)\right]\\ & \;\cdot \left(f-\sqrt{f^{2}+x^{2}+y^{2}}\right)\\ & =const, \end{array}$$

where *∆φ*_*a*,*b*_ *(x, y)* are the phase difference for the different wavelengths of *λ*_*a*_ and *λ*_*b*_, *n*_eff_(*δ*_*a*_, *λ*_*a*,*b*_) and *n*_eff_(*δ*_*b*_, *λ*_*a*,*b*_) are the effective refractive indices along the *x*-axis and *y*-axis direction when the filling ratios are *δ*_*a*_ and *δ*_*b*_ for the different wavelength of *λ*_*a*_ and *λ*_*b*_. *f* is the focal length. According to Eq. (6), the conditions can be achieved by:

(7) $$\begin{array}{ll} & \frac{2\pi }{\lambda_{a}}\left[n_{\text{eff}}\left(\delta_{a},\lambda_{a}\right)-n_{\text{eff}}\left(\delta_{b},\lambda_{a}\right)\right]\\ & \;-\frac{2\pi }{\lambda_{b}}\left[n_{\text{eff}}\left(\delta_{a},\lambda_{b}\right)-n_{\text{eff}}\left(\delta_{b},\lambda_{b}\right)\right]=0, \end{array}$$

As shown in Figure 1(E), the effective refractive index difference between different filling ratios is less sensitive to the wavelength. Therefore, by the precise design of the index profile, the conditions for a chromatic aberration-free GRIN metalens are satisfied over a wide wavelength range. It means that the phase difference of the on-chip GRIN metalens can be eliminated, resulting in a good performance with negligible chromatic aberration [44].

**Figure 2:**
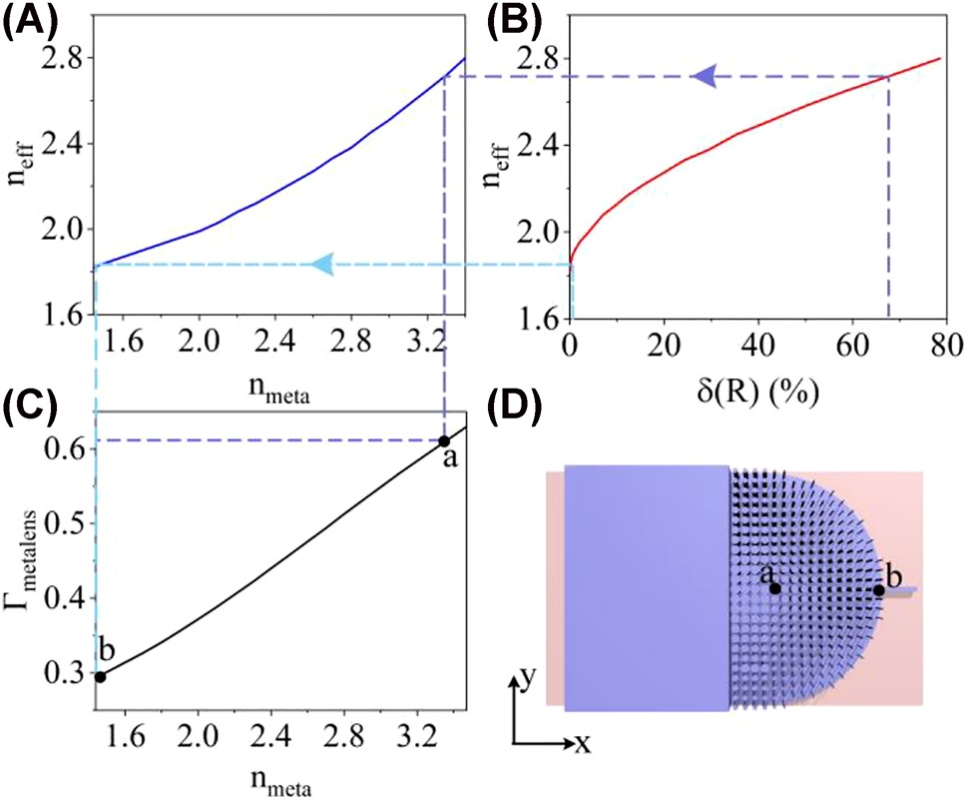
Look-up table about the ability of the GRIN metalens to confine the mode field. (A) Calculated effective refractive index (*n*_eff_) with varied equivalent material index (*n*_meta_). (B) Relationship between effective refractive index (*n*_eff_) and filling ratio (*δ*(*R*)). (C) Calculated fraction Γ_metalens_ of the optical power confined in the 160 nm-height GRIN metalens with varied equivalent material index (*n*_meta_). (D) Location of the maximum (point a) and minimum (point b) refractive index.

To relax the fabrication requirements, the maximum and minimum filling ratios of the silicon nanorods are set to be 67.9 and 0.3%, and the corresponding maximum and minimum refractive indices are 2.72 and 1.86, respectively. It is promising to obtain a low-loss beam transforming with a larger expansion ratio, attributed to the refractive index variation in the *z*-axis direction. Unfortunately, due to the limitation of planar CMOS fabrication process, the index variation in the *z*-axis direction is difficult to be achieved (see Section 2 of the Supplementary Information). Moreover, other materials can be considered to design the on-chip index profile. Silicon or other materials with a high refractive index is a better alternative for the on-chip half Maxwell’s fisheye lens since they can offer a large index contrast between the origin and edge of the lens (see Section 3 of the Supplementary Information). The period of the silicon nanorods is 200 nm and the radius of the GRIN metalens is 4.6 μm. Due to the unique index profile of the on-chip GRIN metalens, the index mismatch between the lens and waveguide will result in a decrease in coupling efficiency. Therefore, tapered silicon nanorods with four columns are adopted to reduce the modal mismatch between the GRIN metalens and the silicon waveguide at the input port (see Section 4 of the Supplementary Information). Although only the xy-plane index profile is designed, the ability of the GRIN metalens to confine the mode field can be varied with the *xy*-plane index profile of the GRIN metalens. The fraction Γ_metalens_ of the optical power confined in the 160 nm-height GRIN metalens is simulated by the finite element method. The look-up table in Figure 2 shows that the fraction Γ_metalens_ of the optical power confined in the GRIN metalens decreases when the filling ratio *δ*(*R*) decreases, resulting in a vertical tapered coupling structure in the *z*-axis direction. Therefore, the optical mode field can be gradually coupled from a 220 nm-height waveguide to a 60 nm-height waveguide. In addition, the design method can be scaled to achieve waveguide coupling with arbitrary widths and heights.

To prove the scalability and fabrication tolerance of the proposed on-chip half Maxwell’s fisheye lens, the lenses with different expansion ratios in width and height, nanorod radius variations, and removal of nanorods with small radius are investigated by the 3D-FDTD method (see Sections 5 and 6 of the Supplementary Information). For different expansion ratios in waveguide widths, the transmission spectra reveal that the insertion losses of <1.6 dB are achieved in a bandwidth of 1100 nm. In the height dimension, the insertion losses of <1.5 dB are maintained in a bandwidth of 650 nm with the input waveguide height varying from 180 to 500 nm. The simulated losses are lower than 1.5 dB in the wavelength range of 1200–2200 nm for the height of the output waveguide varying from 40 nm to 70 nm. When the variations of nanorod radius are below ±20 nm, the simulated 1.5 dB bandwidth can reach 1000 nm. The performance of the GRIN metalens will not be affected and degraded even though the silicon nanorods with diameters below 100 nm are omitted. The on-chip half Maxwell’s fisheye lens exhibits good scalability and high fabrication tolerance.

**Figure 3:**
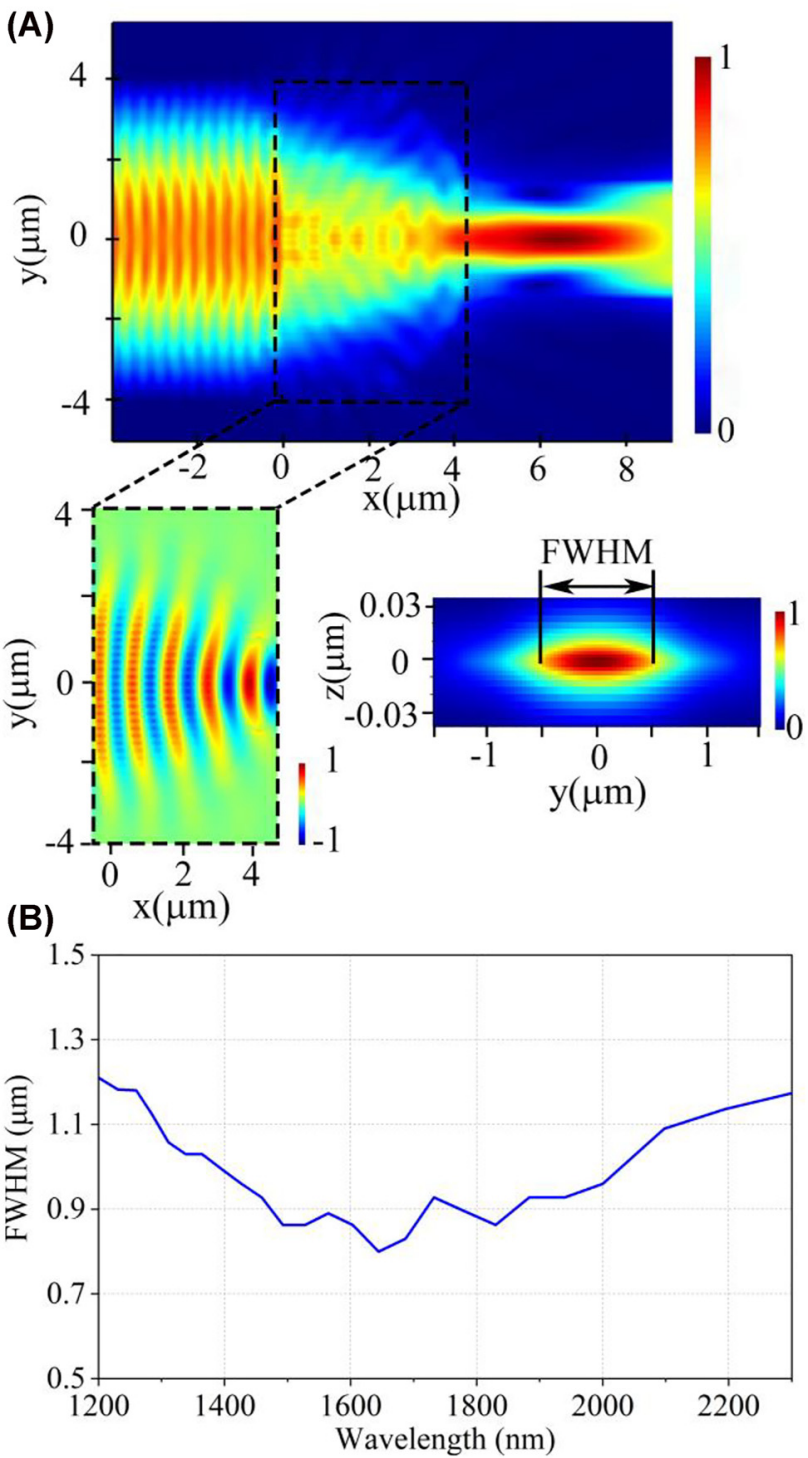
Simulated beam focusing behavior of the on-chip half Maxwell’s fisheye lens. (A) Electrical field distribution of the *xy*-plane view in the lens. Inset: Enlarged electrical field distribution (*E*_*y*_) of the dashed box and the cross-section (*yz*-plane) of the mode profile at the interface between the circumference and the output waveguide. (B) FWHM values of the output mode.

We use the 3D-finite-difference time-domain (3D-FDTD) method to evaluate the on-chip half Maxwell’s fisheye lens. The input light is coupled into an 8 μm-wide waveguide and propagates along the *x*-axis direction. The width of the output waveguide is 3 μm. The beam focusing behavior caused by the wavefront controlled through the GRIN profiles is demonstrated in Figure 3(A). The width of the output mode can be described as full width half maximum (FWHM) in the cross-section, as shown in the inset of Figure 3(A). The FWHM values at the interface between the circumference and the output waveguide are less than 1.2 μm in the operation bandwidth of 1100 nm, as depicted in Figure 3(B), which indicates that the proposed half Maxwell’s fisheye lens exhibits a good beam focusing ability in a large bandwidth.

**Figure 4:**
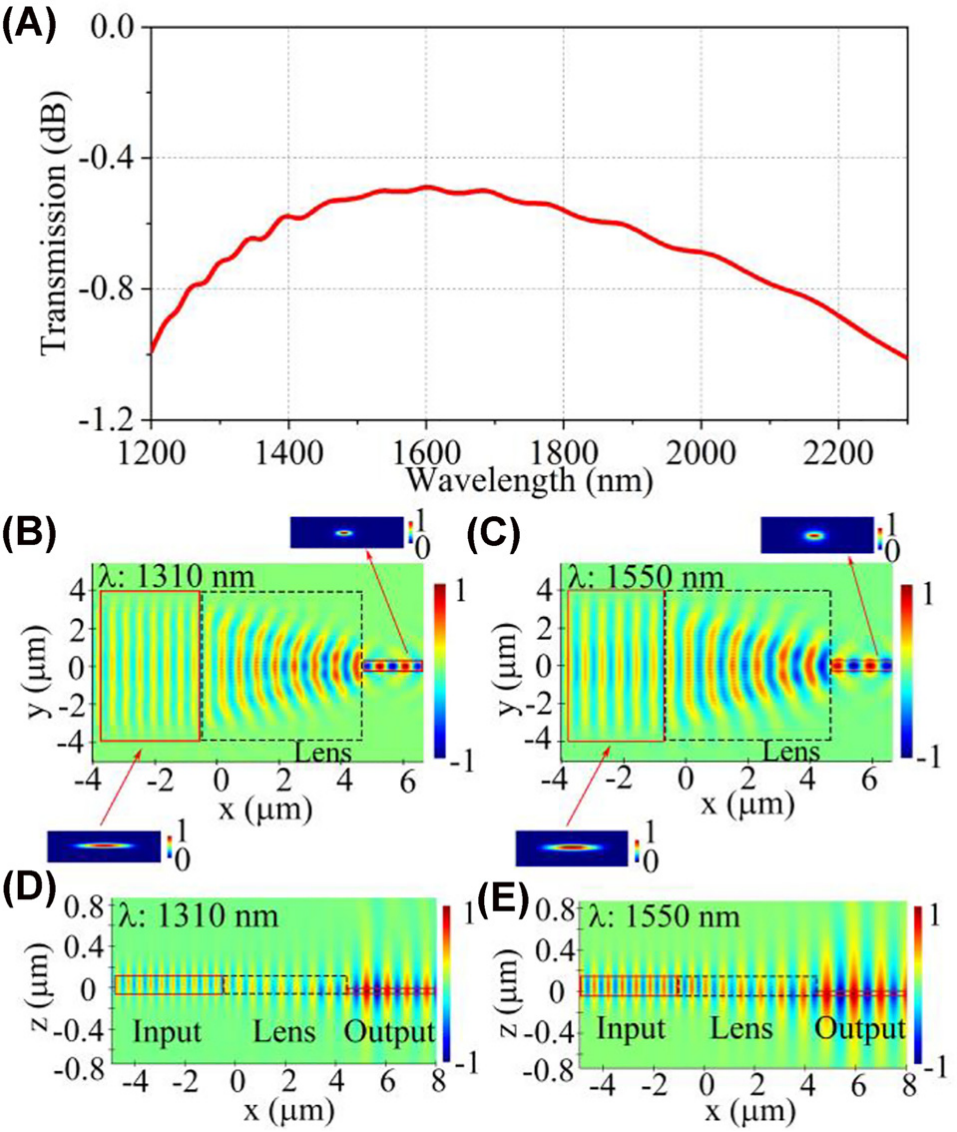
Simulated performance of the on-chip GRIN metalens for matching two modes with different modal sizes. (A) Transmission spectrum of the on-chip GRIN metalens. *Xy*-plane electrical field distribution (*E*_*y*_) of the lens at (B) 1310 nm and (C) 1550 nm. *Xz*-plane electrical field distribution (*E*_*y*_) of the lens at (D) 1310 nm and (E) 1550 nm. Insets display the *yz*-plane TE_0_ mode fields in the input and output silicon waveguides.

Using the index distribution and focusing ability of the on-chip half Maxwell’s fisheye lens, the lens can be utilized in mode coupling with different modal sizes. As an example, a TE fundamental mode in an 8 μm-wide and 220 nm-height silicon waveguide is coupled into a 0.5 μm-wide and 60 nm-height silicon waveguide employing the 5.4 μm-length GRIN metalens. The simulated transmission spectrum is plotted in Figure 4(A), which reveals that the 1 dB bandwidth can reach 1100 nm. Since the maximum and minimum refractive indices of the half Maxwell’s fisheye lens are distributed in the origin and edge of the semicircle, respectively, the proposed on-chip metalens can match two silicon waveguides with different heights and widths with low loss. The field propagation for beam transforming in the height and width dimension at *λ* = 1310 and 1550 nm can be depicted in Figure 4(B)–(E), where Figure 4(D) and (E) show the unique field propagation in the height dimension. When the light from the 60 nm-height, 0.5 μm-wide waveguide spreads through the proposed on-chip GRIN metalens, the optical mode field can be confined to the 220 nm-height, 8 μm-wide waveguide. The beam transforming can be realized for the backward propagation (see Section 7 of the Supplementary Information).

**Figure 5:**
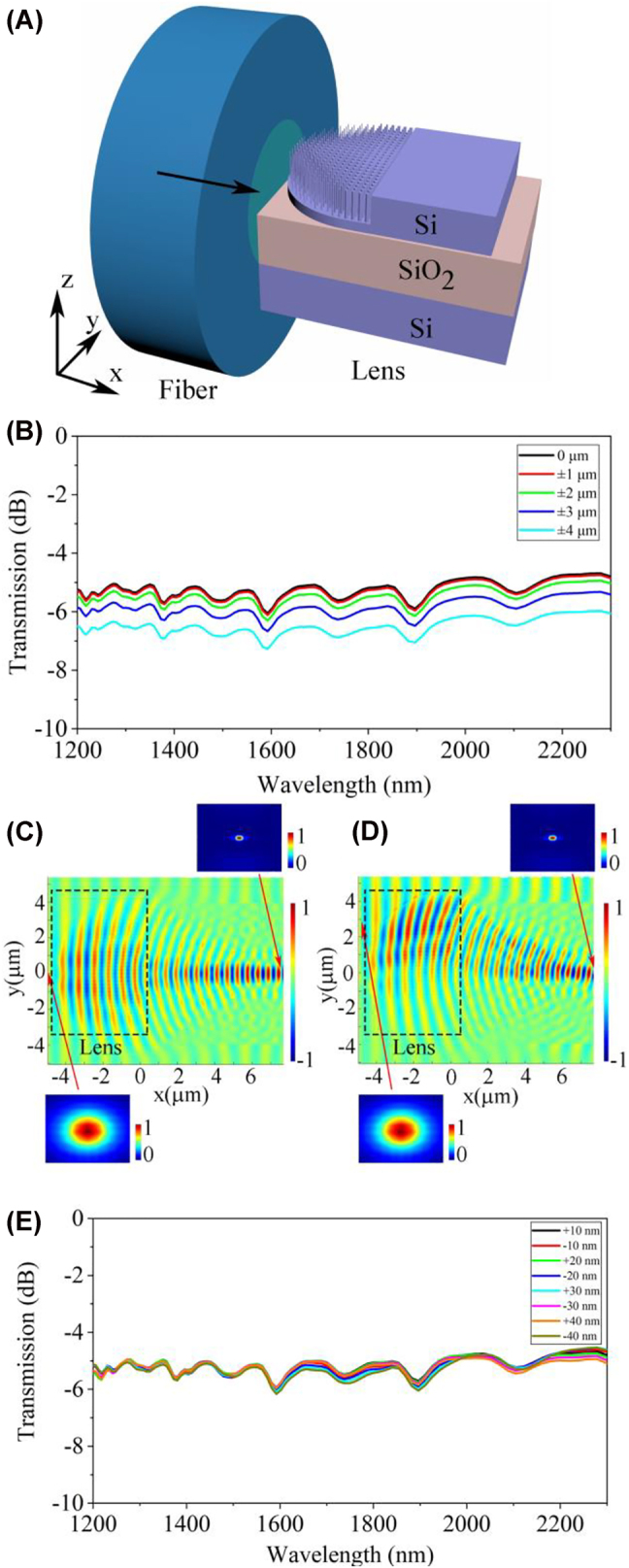
Simulated performance of the on-chip GRIN metalens for fiber-to-chip edge coupling. (A) Schematic of the fiber-to-chip edge coupling device. (B) Simulated coupling responses of the on-chip GRIN metalens for different relative positions of the fiber. (C) *Xy*-plane electrical field distribution (*E*_*y*_) of the on-chip devices when the relative position of the fiber is not shifted. (D) *Xy*-plane electrical field distribution (*E*_*y*_) of the on-chip devices when the relative position of the fiber is shifted by 3 μm in the *y*-axis direction. Insets display the *yz*-plane TE_0_ mode fields in the fiber and the silicon waveguide. (E) Simulated coupling responses of the on-chip GRIN metalens for different nanorods radius variations.

In addition to on-chip waveguide interconnecting with low loss and large operation bandwidth, robust coupling between photonics structures with very different dimensions can be realized by the on-chip GRIN metalens, such as fiber-to-chip edge coupling. The misalignment sensitivity of the GRIN metalens for fiber-to-chip edge coupling is investigated by the 3D-FDTD method. The schematic structure and simulated coupling responses of the device are shown in Figure 5(A) and (B). A fiber with a mode field diameter of 4.1 μm is directly aligned to the spherical circumference. The variation of misalignment loss can be maintained within 1 dB by shifting the relative position of the fiber from −3 μm to + 3 μm in the *y*-axis direction. Although the relative position of the fiber is shifted by 3 μm in the *y*-axis direction, benefitting from the control of the wavefront phase by the index profile, a stable guided-mode transmission can still be eventually formed in the waveguide, as depicted in Figure 5C and D. Therefore, for fiber-to-chip edge coupling, the beam focusing property of the on-chip lens is less sensitive to the location of input lights, which indicates that the on-chip lens exhibits higher misalignment tolerance. As a comparison, the conventional edge coupler exhibits an alignment tolerance of approximately ±0.9 μm in the *y*-direction for a 1 dB excess loss for a fiber with a mode-field diameter of 4.1 μm [45]. To investigate the fabrication tolerance of the on-chip GRIN metalens for fiber-to-chip edge coupling, the coupling responses of the on-chip GRIN metalens are simulated with the varied nanorods radius. As shown in Figure 5(E), the coupling responses remain unchanged in the wavelength range of 1200–2300 nm when the nanorods radius variations are below ±40 nm. Various reported fiber-to-chip edge couplers are summarized in Table 1, which are utilized to match the modal sizes between the fiber and the waveguide. Compared to previous work, the proposed GRIN metalens exhibits a compact footprint and ultrabroad bandwidth for fiber-to-chip edge coupling. Moreover, designed fiber assembly and commercial matching oils can be used to improve the coupling efficiency [46].

**Table 1:** Performances of various fiber-to-chip edge couplers.

Architecture	Length	Loss	Bandwidth
	(μm)	(dB)	(nm)
4-tips tapers [14]	90	0.42	415
Trident-shape tapers [15]	35	2	100
Metamaterial tapers [16]	90	3	120
Multilayer tapers [17]	260	0.42	150
Waveguide array [18]	500	1	300
Luneburg lens [19]	30	6.5	
Multilayer GRIN lens [20]	11.8	2.6	50
Asymmetric GRIN lens [21]	20	0.45	90
Micro-GRIN lens [22]	10.5	1.5	
This work	**5.4**	5.3	**1100**

Bold values indicate the outstanding features in our work.

## 3 Device fabrication and characterization

**Figure 6:**
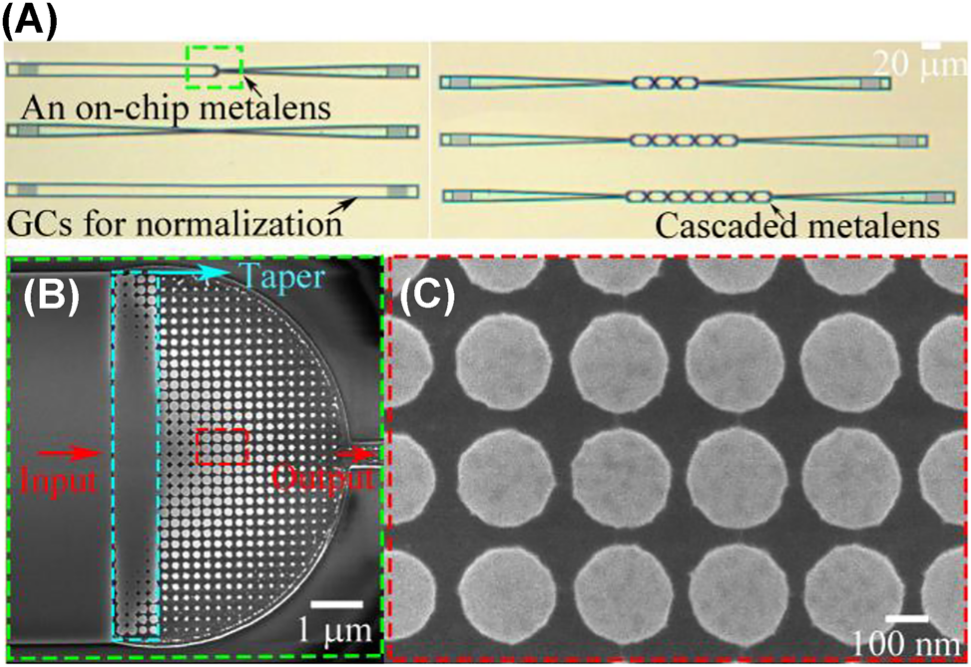
Photos of the fabricated on-chip GRIN metalens. (A) Optical microscope graphs of an on-chip half Maxwell’s fisheye lens, cascaded half Maxwell’s fisheye lenses, and grating couplers. (B) SEM photo of the fabricated on-chip half Maxwell’s fisheye lens. (C) Magnified SEM image of the periodic nanorod arrays. GCs, Grating couplers.

The proposed on-chip half Maxwell’s fisheye lens was manufactured on an SOI wafer with a top silicon thickness of 220 nm and a buried oxide thickness of 3 μm. The designed patterns including grating couplers, silicon nanorods, and waveguides were defined on the resist (AR-P 6200.09) employing electron-beam lithography (Vistec EBPG 5200^+^). After chemical development, inductively coupled plasma (ICP) dry etching (SPTS DRIE-I) was utilized to etch 70 nm for the grating couplers, 160 nm for the nanorods, and 220 nm for the waveguides. Consequently, a 1 μm-thick silica upper cladding was deposited through plasma-enhanced chemical vapor deposition (PECVD, Oxford). The optical microscope graphs and scanning electron microscope (SEM) images of the on-chip GRIN metalens are given in Figure 6.

To evaluate the large bandwidth of the on-chip GRIN metalens in the experiment based on the spectrum scanning systems, the grating couplers with four periods are fabricated to couple the TE_0_ mode into or out of the waveguides, including a period of 505 nm for the wavelength range of 1280–1350 nm, a period of 530 nm for the wavelength range of 1350–1440 nm, a period of 570 nm for the wavelength range of 1440–1510 nm and a period of 630 nm for the wavelength range of 1510–1620 nm (see Section 8 of the Supplementary Information). In the simulation, a 90° bending and an adiabatic taper are introduced at the input port to ensure that the on-chip devices are not on the same beam axis. The simulated transmission spectrum of the device with the bending waveguide is similar to that of the device without the 90° bending waveguide (see Section 9 of the Supplementary Information). It indicates that the beam is indeed converted into the narrow waveguide by the on-chip lens and does not exist in free-space spatial modes. Tilted angles of the fiber probes are modified to characterize ultra-broadband devices due to the limited bandwidth of the grating couplers [30, 47]. To maximize the bandwidth of the grating coupler in the experiment, two tilted angles of ∼14° and ∼5° that the vertical coupling setup in our lab can support are used to test the devices, respectively.

## 4 Experimental results

**Figure 7:**
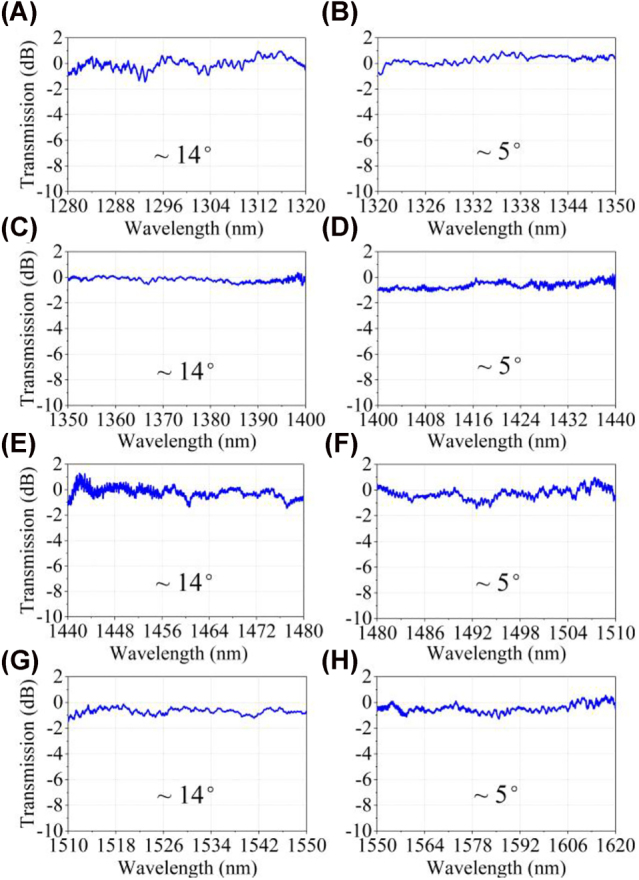
Experimental transmission spectra of the on-chip GRIN metalens. The transmission spectra in the wavelength ranges of (A) 1280–320 nm (C) 1350–1400 nm, (E) 1440–1480 nm, and (G) 1510–1550 nm are tested with a tilted angle of ∼14°. The transmission spectra in the wavelength ranges of (B) 1320–1350 nm (D) 1400–1440 nm, (F) 1480–1510 nm, and (H) 1550–1620 nm are measured with a tilted angle of ∼5°.

To obtain the performance of the fabricated on-chip GRIN metalens, the transmission spectra of an on-chip GRIN metalens in the experiment are normalized by the spectra of the grating couplers. Covering the wavelength range of 1280–1620 nm, the insertion loss of an on-chip half Maxwell’s fisheye lens is below 1 dB, as shown in Figure 7. The 1 dB operation bandwidth in the experiment is only 340 nm due to the lack of laser sources with other wavelengths. The measured low-loss performance indicates a TE fundamental mode output at the narrow waveguide port with high purity. The fluctuations in the transmission response are related to the flaws introduced in the manufacturing process.

**Figure 8:**
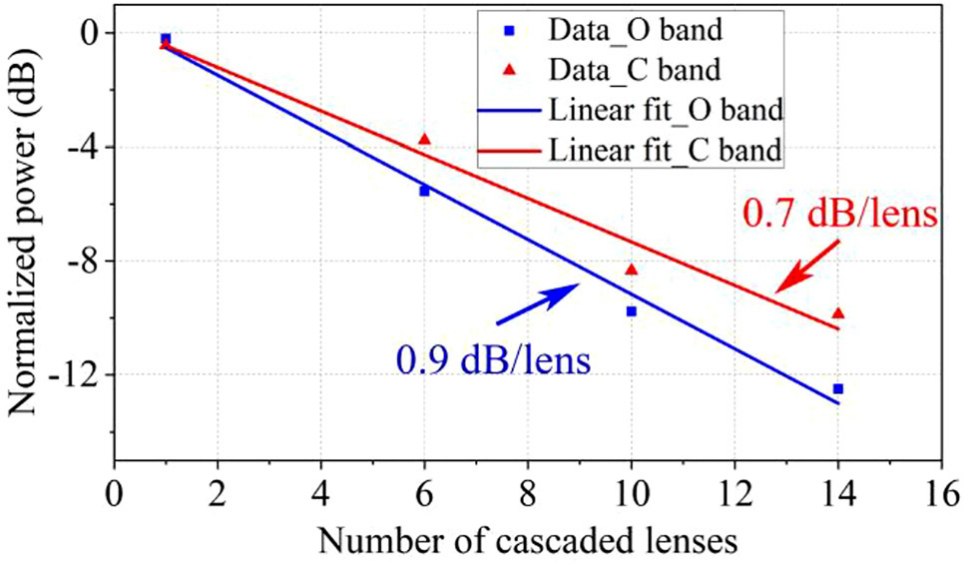
Insertion losses of cascaded on-chip half Maxwell’s fisheye lenses.

We also fabricate 6, 10, and 14 cascaded lenses on the same chip to achieve more accurate loss values. By linearly fitting the number of lenses and the normalized output powers, the insertion losses of beam transforming in two waveguides with different heights and widths are 0.9 and 0.7 dB at 1310 and 1550 nm for an on-chip GRIN metalens, respectively, as shown in Figure 8. As a comparison, the insertion losses of the 5.4 μm-length GRIN metalens for beam transforming in two waveguides with different heights and widths are comparable with a conventional adiabatic tapered waveguide with a length of 150 μm [26].

Considering the width expansion ratio *ω*_in_ */ω*_out_ and height expansion ratio *h*_in_ */h*_out_, insertion loss, operation bandwidth, and length, the performance of the beam transforming are summarized in Table 2. These advanced devices are implemented for beam transforming in the waveguides with different heights and widths. In [23, 49], SU-8 material is introduced to construct height-variant waveguides. In [48], the height-variant waveguides are fabricated by the complex etching process. In [50, 51], mode filed coupling in height dimension is achieved by multilayer waveguides. Compared to previous works, the demonstrated on-chip half Maxwell’s fisheye lens exhibits ultrabroad bandwidth, large expansion ratio, low insertion loss, and compact footprint.

**Table 2:** Performances of various devices with beam transforming in two waveguides with different heights and widths.

Architecture	*ω*_out_ (μm)	*ω*_in_ (μm)	*h*_out_ (μm)	*h*_in_ (μm)	Expansion ratio	Length (μm)	Loss (dB)	Bandwidth (nm)
3D SU-8 taper [23]	4	10	1	10	25	850	2.8	40
3D taper [48]	1	6	1	6	36	71.4	0.48	–
3D polymer taper [49]	5	10	1	10	20	850	3	70
Buried 3D taper [50]	0.45	3	0.22	1.5	45	50	0.56	35
Hybrid SON taper [51]	0.45	1	0.22	0.68	3	30	0.02	135
This work^a^	0.5	8	0.06	0.5	**133**	**5.4**	< 1.5	**1100**
This work	0.5	8	0.06	0.22	**59**	**5.4**	< 1	**340**

^a^Simulation result. Bold values indicate the outstanding features in our work.

## 5 Conclusions

We have demonstrated an integrated half Maxwell’s fisheye lens on an SOI substrate, which enables beam transforming in two waveguides with different heights and widths. The effective refractive index distribution required by the GRIN metalens is mapped to the periodic silicon nanorods array. In the simulation, an expansion ratio in two waveguides with different heights and widths is 133 enabled by the on-chip 5.4 μm-length half Maxwell’s fisheye lens. The beam transforming can be realized between a 500 nm-height, 8-μm-wide waveguide and a 60 nm-height, 0.5 μm-wide waveguide. The simulated 1 dB operation bandwidth can reach 1100 nm. The measured losses of the fabricated beam transforming device are less than 1 dB in the wavelength range of 1280–1620 nm. Good scalability and high fabrication tolerance of the GRIN metalens are validated. The on-chip half Maxwell’s fisheye lens can also be utilized for fiber-to-chip coupling to achieve high alignment tolerance. We believe that our experimental demonstration of the on-chip half Maxwell’s fisheye lens opens up a promising direction for GRIN metalens-based on-chip transforming, which is distinguished from conventional spot size converters or adiabatic tapers.

## Supplementary Material

Supplementary Material Details
