# Supplementary material for: Ultra-broadband on-chip beam focusing enabled by GRIN metalens on silicon-on-insulator platform
Source: Nanophotonics. 2022 Jul 14;11(16):3603–12. doi: 10.1515/nanoph-2022-0242 (PMC11501878; doi:10.1515/nanoph-2022-0242)
Supplement: Supplementary file 1 — Supplementary Material Details [file j_nanoph-2022-0242_suppl.docx]

Supplementary Information for:

**Ultra-broadband on-chip beam focusing enabled by GRIN metalens on silicon-on-insulator platform**

Jian Shen^1^, Yong Zhang^1,*^, Yihang Dong^1^, Zihan Xu^1^, Jian Xu^2^, Xueling Quan^2^, Xihua Zou^3^, and Yikai Su^1^

1 State Key Lab of Advanced Optical Communication Systems and Networks, Department of Electronic Engineering, Shanghai Jiao Tong University, Shanghai 200240, China

2 Center for Advanced Electronic Materials and Devices, Shanghai Jiao Tong University, Shanghai 200240, China

3 Center for Information Photonics and Communications, School of Information Science and Technology, Southwest Jiao Tong University, Chengdu 611756, China

*E-mail: yongzhang@sjtu.edu.cn

**Content**

[1 Anisotropic property of the on-chip GRIN metalens 2](#_Toc107049018)

[2 3D lens design 2](#_Toc107049019)

[3 Materials for the on-chip GRIN metalens 4](#_Toc107049020)

[4 Efficiency improvement 4](#_Toc107049021)

[5 Scalability of the half Maxwell’s fisheye lens 5](#_Toc107049022)

[6 Fabrication tolerance 6](#_Toc107049023)

[7 Backward propagation 7](#_Toc107049024)

[8 Measurement setup and grating couplers 8](#_Toc107049025)

[9 Characterization with 90° bending waveguide 8](#_Toc107049026)

[References 9](#_Toc107049027)

# 1 Anisotropic property of the on-chip GRIN metalens

For the TE polarization, the subwavelength silicon nanorods exhibit an anisotropic property. We assume that the local equivalent refractive index at any nanorods waveguide along the y-axis direction is given by the equivalent refractive index of a y-invariant silicon nanorods array with the same period and filling ratio δ. The structure can be approximated by a homogeneous anisotropy metamaterial with diagonal index tensors n(δ) = diag[n_xx_(δ), n_yy_(δ), n_zz_(δ)] [1]. The equivalent material indices of anisotropic metamaterial are given by [2, 3]:

$\begin{matrix} n_{\text{||}}(R)^{2}=\delta(R)\cdot n_{Si}^{2}+\left[1-\delta(R)\right]\cdot n_{SiO_{2}}^{2} \\ \frac{1}{n_{\text{⊥}}(R)^{2}}\text{=}\delta(R)\cdot\frac{1}{n_{Si}^{2}}\text{+}\left[1-\delta(R)\right]\cdot\frac{1}{n_{SiO_{2}}^{2}} \end{matrix}$, (S1)

where n_||_(R) and n_⊥_(R) are the equivalent material indices for polarization parallel (along the y-axis direction) and perpendicular (along the z-axis direction) to the xy plane, respectively. The diagonal material index tensors for the anisotropy material can be written as [4]:

$n=\text{diag }[n_{\text{xx}},n_{yy},n_{zz}]\text{ }=\text{diag }[n_{||},n_{||},n_{\text{⊥}}]$ (S2)

where n_yy_ is consistent with the GRIN [1]. As shown in Fig. S1, the metalens based on the GRIN profile exhibits anisotropic characteristics, but this anisotropy will not affect the focusing property.


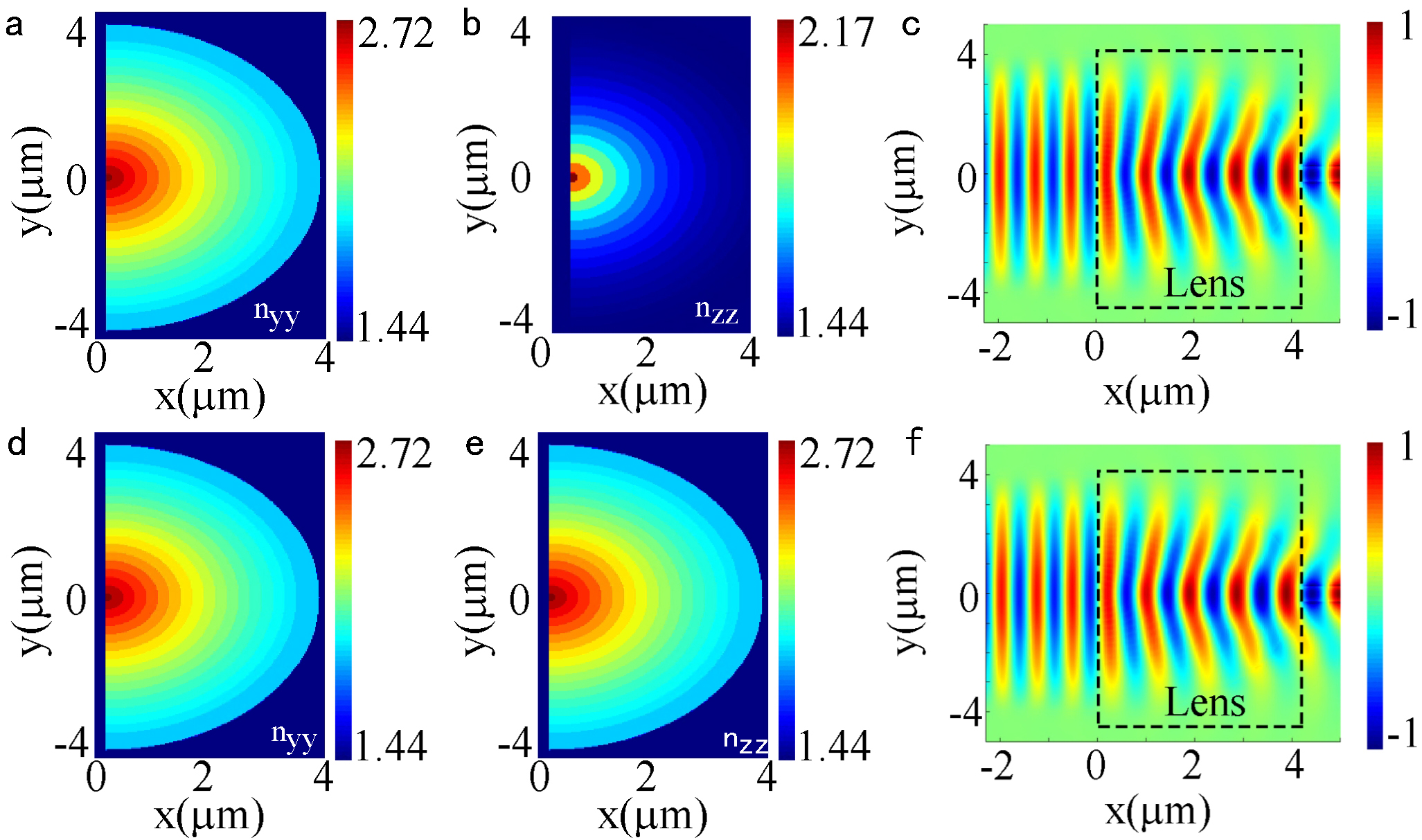


**Figure S1:** Anisotropic lens and isotropic lens. (a) n_yy_ and (b) n_zz_ index profile of the anisotropic lens. (c) Electrical field distribution (Ey) of the anisotropic lens. (d) n_yy_ and (e) n_zz_ index profile of the isotropic lens. (c) Electrical field distribution (Ey) of the isotropic lens.

# 2 3D lens design

The refractive index variation in the z-axis direction of the on-chip lens provides a different perspective for designing the on-chip lens. In the simulation, a conventional hemisphere with a radius of 4.6 μm is used to construct the refractive index variation in the z-axis direction of the 3D lens, as presented in Fig. S2(a). The index profiles of the yz plane at x=0 μm and x=2 μm are plotted in Fig. S2(b) and (c). The 3D lens is employed to connect an 8-μm-wide, 2-μm-height and a 0.5-μm-wide, 60-nm-height waveguide. As shown in Fig. S2(d), a beam transforming for TE fundamental mode can be achieved between an 8-μm-wide, 2-μm-height waveguide, and a 0.5-μm-wide, 60-nm-height waveguide. Benefiting from the chromatic aberration-free imaging property of the 3D lens, the simulated losses remain lower than 0.5 dB in the wavelength range of 1130 nm ~ 3000 nm, as depicted in Fig. S2(e). Compared to the 2D lens, a broader operation bandwidth and lower losses are achieved for the 3D lens. In addition, it is promising to obtain a low-loss beam transforming with a larger expansion ratio, attributed to the refractive index variation in the z-axis direction. Unfortunately, due to the limitation of planar CMOS fabrication process, the index variation in the z-axis direction is difficult to be achieved.


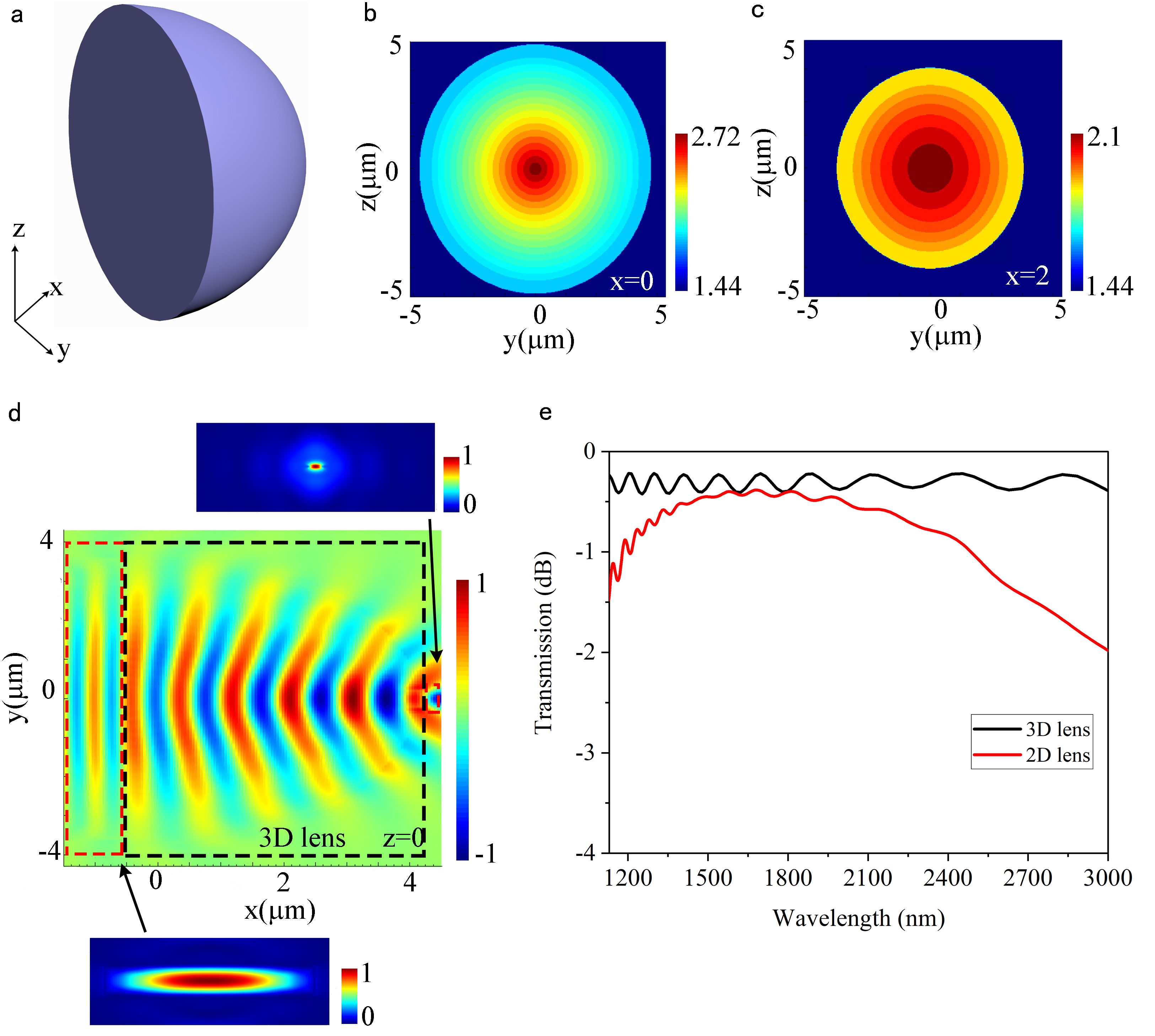


**Figure S2:** (a) Illustration of the 3D lens. Index profile in the yz plane when (b) x=0 μm, and (c) x=2 μm. (d) Xy-plane electrical field distribution (E_y_) of the 3D lens. (e) Simulated transmission spectrum of the 2D lens and 3D lens. Insets display the yz-plane mode fields in the waveguides.

# 3 Materials for the on-chip GRIN metalens

The material system is one of the important directions in the development of integrated devices, so the materials for the on-chip GRIN metalens are discussed. Silicon nitride (SiN) is a new promising material with a refractive index of ~2 for integrated photonics due to its wide optical bandgap and ultra-low absorption loss. Therefore, SiN instead of Si is adopted to simulate the on-chip GRIN metalens. Due to the small index of SiN, the thickness of the waveguide should be increased to support guided mode in the on-chip GRIN metalens. Therefore, the effective refractive index n_eff_(R) in the metamaterial layer with 200-nm-height SiN slab and 160-nm-height SiN nanorods is calculated by the finite element method. As shown in Fig. S3, SiN integrated platform offers a smaller effective refractive index range for the metamaterial layer, which is difficult to meet the requirement of the index profile for the on-chip half Maxwell’s fisheye lens. Therefore, silicon or other materials with a high refractive index is a better alternative for the on-chip half Maxwell’s fisheye lens since they can offer a large index contrast between the origin and edge of the lens.


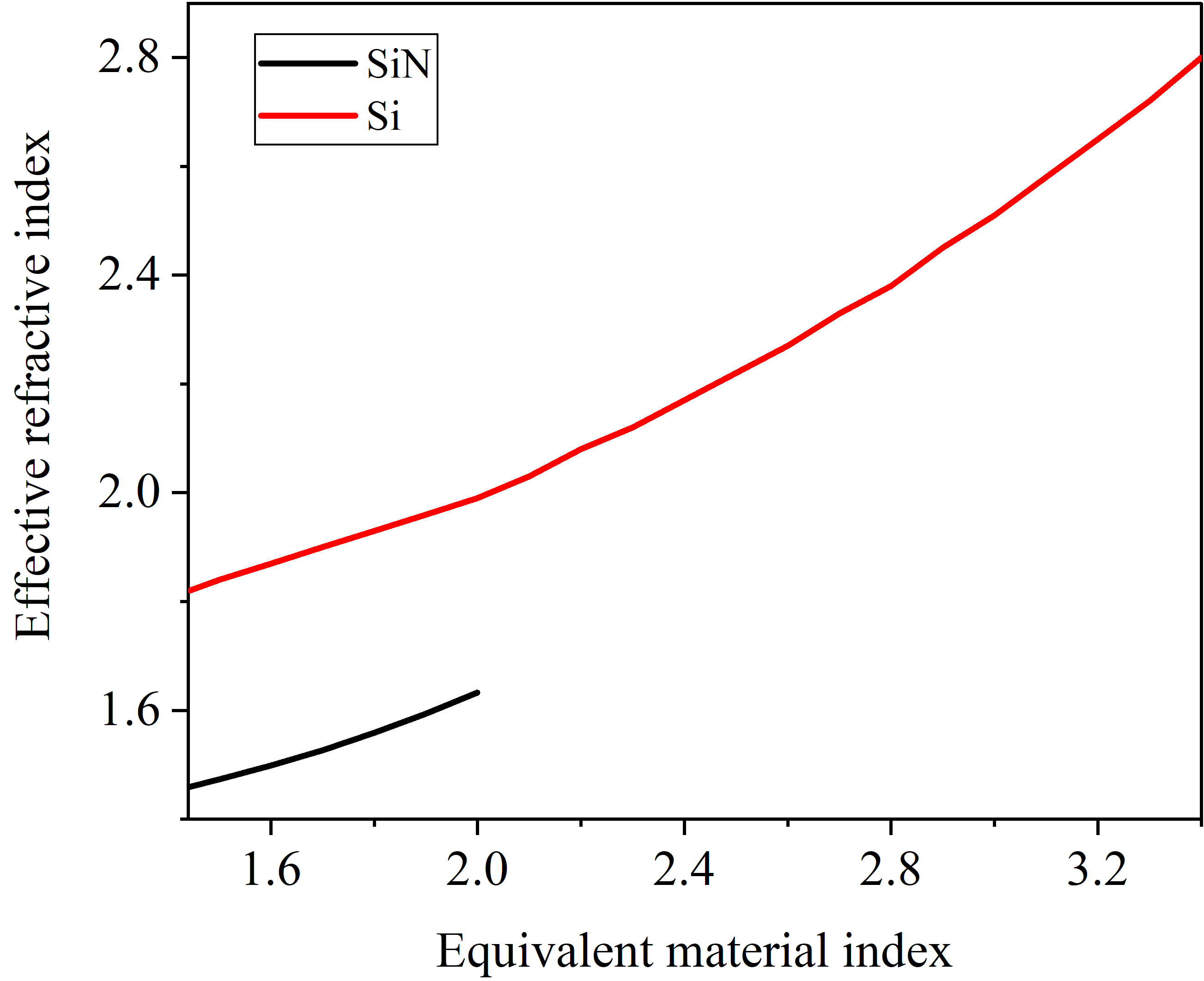


**Figure S3:** Calculated effective index of the metamaterial layer as a function of the material index of SiN and Si.

# 4 Efficiency improvement

The effective refractive index of the on-chip GRIN metalens changes from 1.86 to 2.72, as depicted in Fig. 1(A). The maximum and minimum refractive indices of the half Maxwell’s fisheye lens are distributed at the origin and edge of the semi-circle, respectively. Since the effective refractive index of the input waveguide is constant in the y-axis direction, the index mismatch between the GRIN metalens and waveguide will result in a decrease in coupling efficiency. In the design, tapered silicon nanorods with four columns are added to reduce the modal mismatch between the GRIN metalens and the silicon waveguide at the input port. The period of the tapered nanorods array is the same as the nanorods array of the GRIN metalens. As plotted in Fig. S4(a), ∆r_1_ denotes the variation of radius for the columns of #1, #2 and ∆r_2_ denotes the variation of radius for the columns of #3, #4. The coupling efficiency can be improved well by increasing the radius variation to 60 nm for the columns of #1, #2 and the radius variation to 40 nm for the columns of #3, #4, as shown in Fig. S4(b).


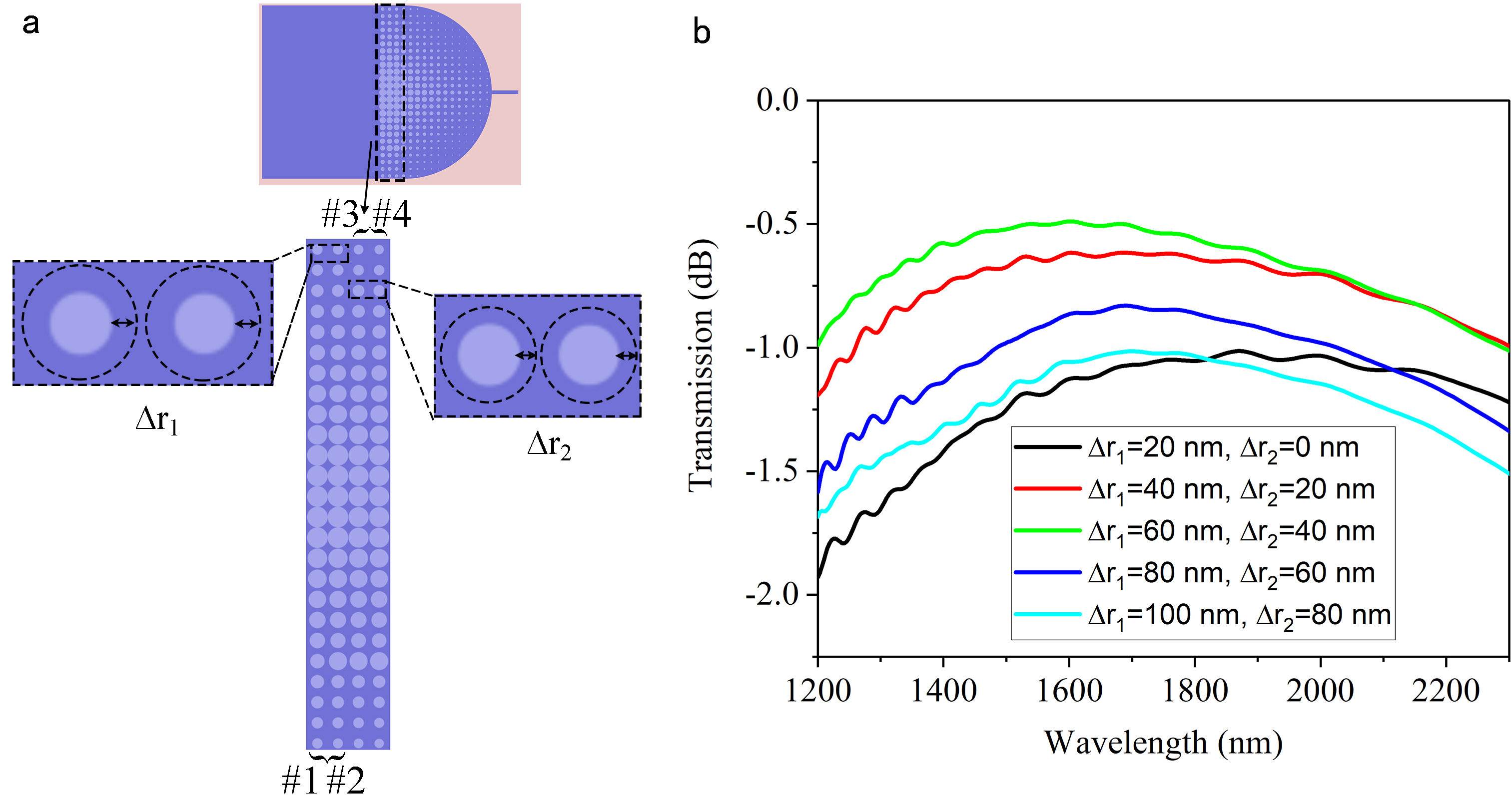


**Figure S4:** Tapered silicon nanorods array for efficiency improvement. (a) Schematic of the tapered silicon nanorods array. (b) Simulated transmission spectra with different radius variations.

# 5 Scalability of the half Maxwell’s fisheye lens

The scalability of the on-chip half Maxwell’s fisheye lens is verified by 3D FDTD methods. The transmission spectra of the lenses with different expansion ratios are shown in Fig. S5. The simulated insertion losses can maintain lower than 1.6 dB in the wavelength range of 1200 nm ~ 2300 nm with the width of the input waveguide ranging from 4 μm to 9 μm. When the width of the output waveguide varies from 300 nm to 1100 nm, the simulated insertion losses can be lower than 1.4 dB in the wavelength range of 1200 nm ~ 2300 nm. For the height of the input waveguide varying from 180 nm to 500 nm, the maximum height expansion ratio can reach 1:8, and the losses are lower than 1.5 dB in the wavelength range of 1350 nm ~ 2000 nm. The simulated losses are lower than 1.5 dB in the wavelength range of 1200 nm ~ 2200 nm for the height of the output waveguide varying from 40 nm to 70 nm.


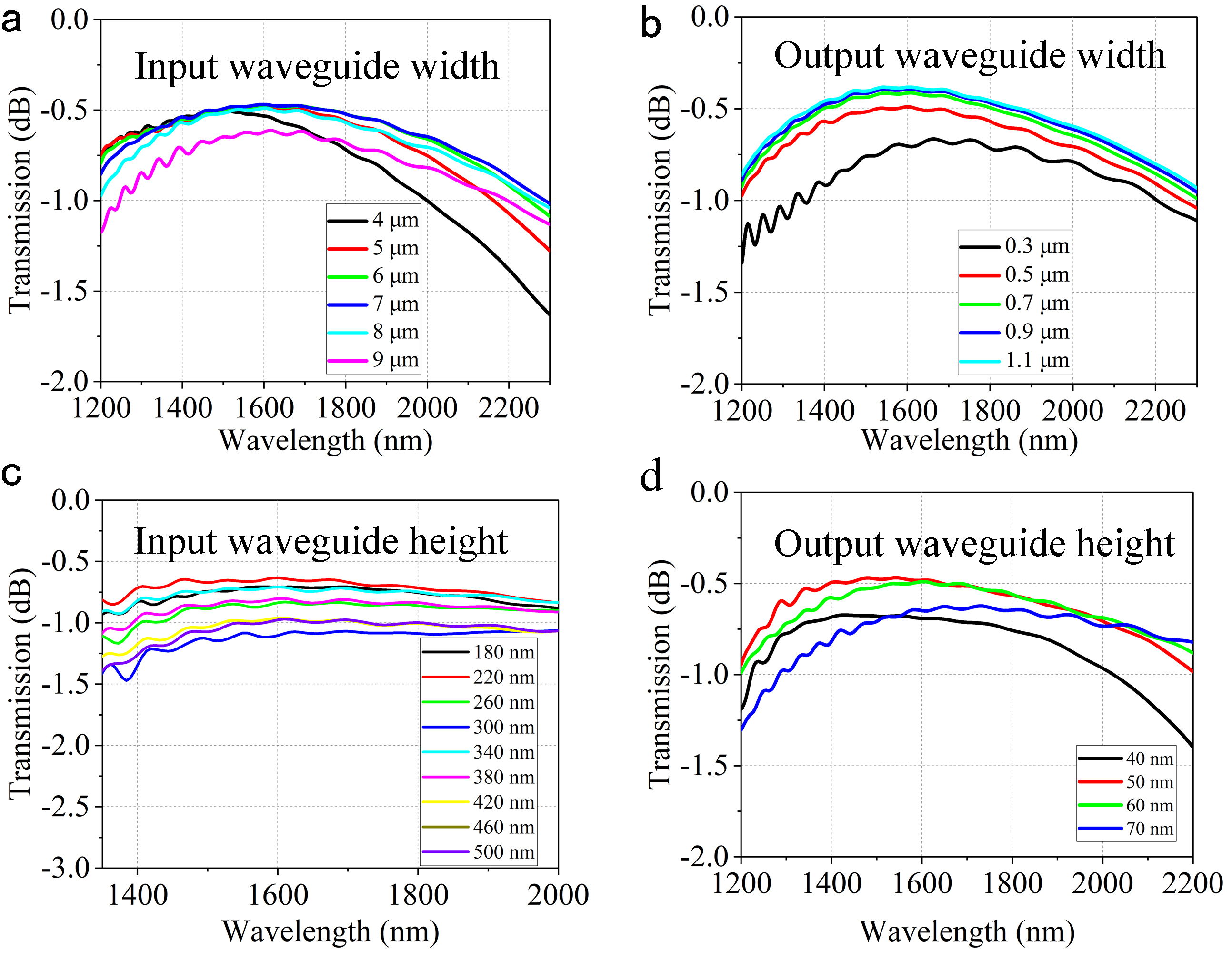


**Figure S5:** Scalability of the proposed on-chip half Maxwell’s fisheye lens. Simulated transmission spectra in the wavelength range of 1200 nm ~ 2300 nm for (a) input waveguide width variation and (b) output waveguide width variation. Simulated transmission spectra in the wavelength range of 1350 nm ~ 2000 nm for different heights of (c) the input waveguide and (d) the output waveguide.

# 6 Fabrication tolerance

The radius variation of periodic silicon nanorods is simulated by 3D FDTD methods. As shown in Fig. S6(a), the insertion losses are lower than 1.5 dB in the wavelength range of 1300 nm ~ 2300 nm with a radius variation of ± 20 nm. For the proposed on-chip metalens, the minimum nanorod radius is 6 nm. The small-radius nanorods are challenging in the fabrication process. The silicon nanorods with a radius of < 50 nm are removed in the simulation and the insertion losses can maintain lower than 1.1 dB in the wavelength range of 1200 nm ~ 2300 nm, as shown in Fig. S6(b). The simulated results indicate that the on-chip half Maxwell’s fisheye lens exhibits high fabrication tolerance.


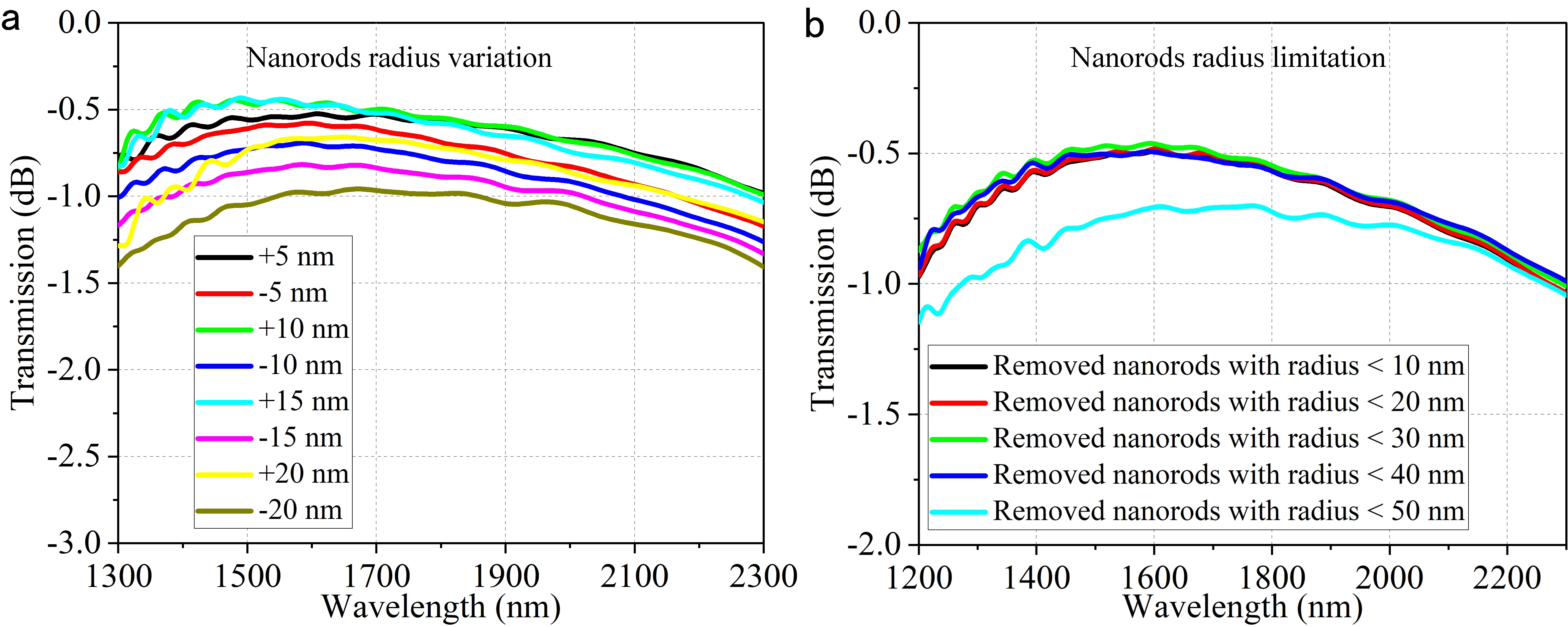


**Figure S6:** Fabrication tolerance of the on-chip half Maxwell’s fisheye lens. (a) Simulated transmission spectra in the wavelength range of 1300 nm ~ 2300 nm with the silicon nanorods radius variation. (b) Simulated transmission spectra in the wavelength range of 1200 nm ~ 2300 nm with the silicon nanorods radius limitation.

# 7 Backward propagation

When the light from the 60-nm-height, 0.5-μm-wide waveguide spreads through the proposed on-chip GRIN metalens, the optical mode field can be confined to the 220-nm-height, 8-μm-wide waveguide. The beam transforming can be realized for the backward propagation. As shown in Fig. S7, the simulated transmission spectrum and mode field distribution of backward propagation are similar to those of the forward propagation.


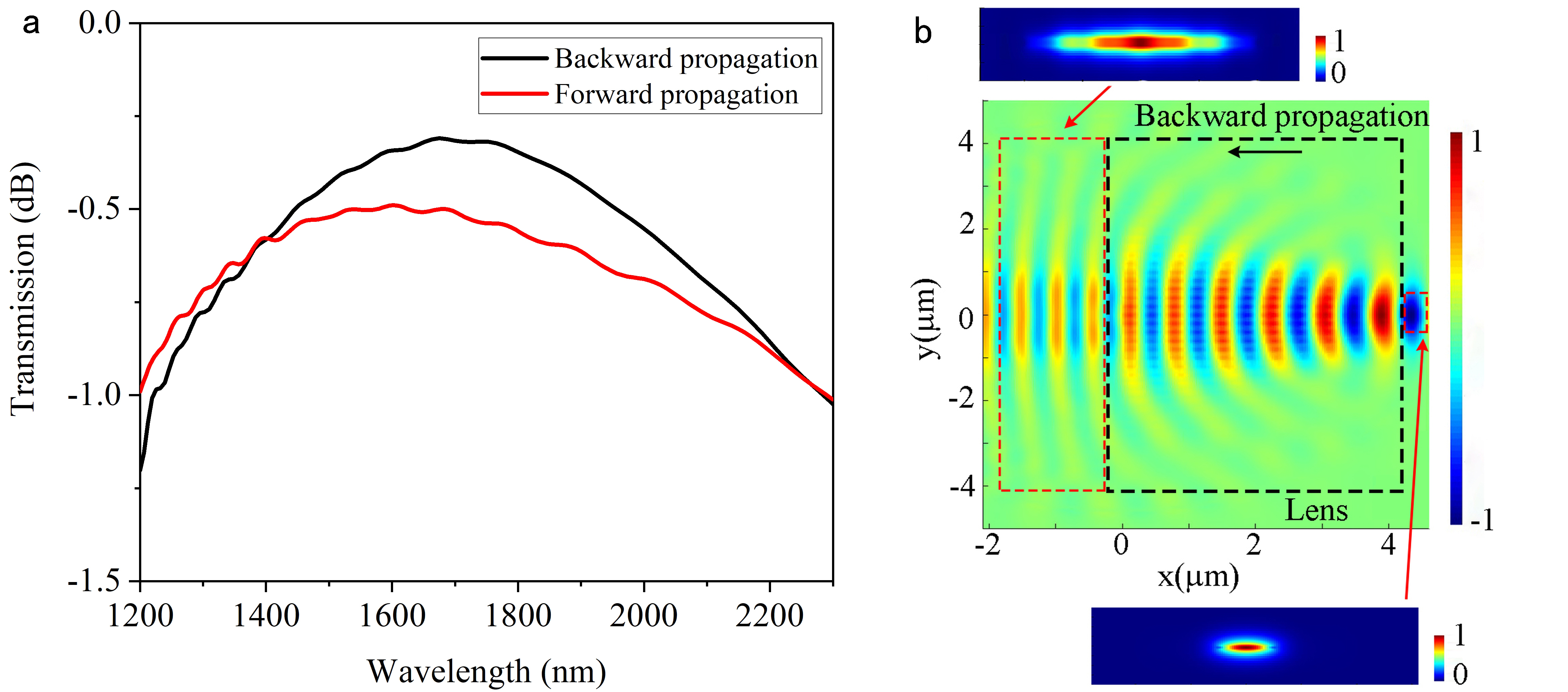


**Figure S7:** Backward propagation for the on-chip GRIN metalens. (a) Simulated transmission spectra for the backward propagation. (b) Simulated field propagation for the backward propagation. Insets display the yz-plane mode fields in the waveguides.

# 8 Measurement setup and grating couplers

To verify device performance accurately, multiple groups of grating couplers with four different periods are fabricated on the same chip. Among them, two kinds of grating couplers with the same period are fabricated: one kind of grating coupler is connected with a 220-nm-height waveguide, and the other is connected with a 60-nm-height waveguide. Due to the limited bandwidth of the on-chip grating couplers, the optical fiber probes with two tilted angles are used to characterize ultra-broadband devices. By referring to the optical power coupled out of the chip, the vertical coupling platform is adjusted to align the optical fiber probes with the on-chip grating couplers. The experiment setups for the wavelength ranges of 1280 nm ~ 1350 nm and 1510 nm ~ 1620 nm consist of two tunable continuous-wave lasers (Santec TSL-550 and Keysight 81960A), associated with an optical power meter (Keysight N7744A). For the remaining wavelength bands, a wideband optical source module associated with an optical spectrum analyzer (YOKOGAWA AQ6370C) is employed to characterize the devices in the wavelength range of 1350 nm ~ 1510 nm. Fig. S8 shows the transmission spectra of the fabricated grating couplers with four different periods of 505 nm, 530 nm, 570 nm, and 630 nm, respectively. The duty cycles of the grating couplers are 50%.





**Figure S8:** Measured transmission spectra of the fabricated grating couplers on the same chip.

# 9 Characterization with 90° bending waveguide

To validly characterize the on-chip GRIN metalens, a 90° bending waveguide and an adiabatic taper are introduced at the input port to ensure that the on-chip devices are not on the same beam axis. In the simulation, the beam propagates from the y-axis direction to the x-axis direction through a 90° bending waveguide with a radius of 20 μm, a width of 1 μm, and a thickness of 220 nm. A 40-μm-length adiabatic taper is employed to convert the 1-μm-wide, 220-nm-height waveguide to an 8-μm-wide, 220-nm-height waveguide, which is the input waveguide of the on-chip GRIN metalens. As shown in Fig. S9, the simulated transmission spectrum of the device with the bending waveguide is similar to that of the device without the 90° bending waveguide. The low excess losses are attributed to the propagation loss of the bending waveguide and adiabatic taper. It indicates that the beam is indeed converted into the narrow waveguide by the on-chip lens and does not exist in free-space spatial modes.


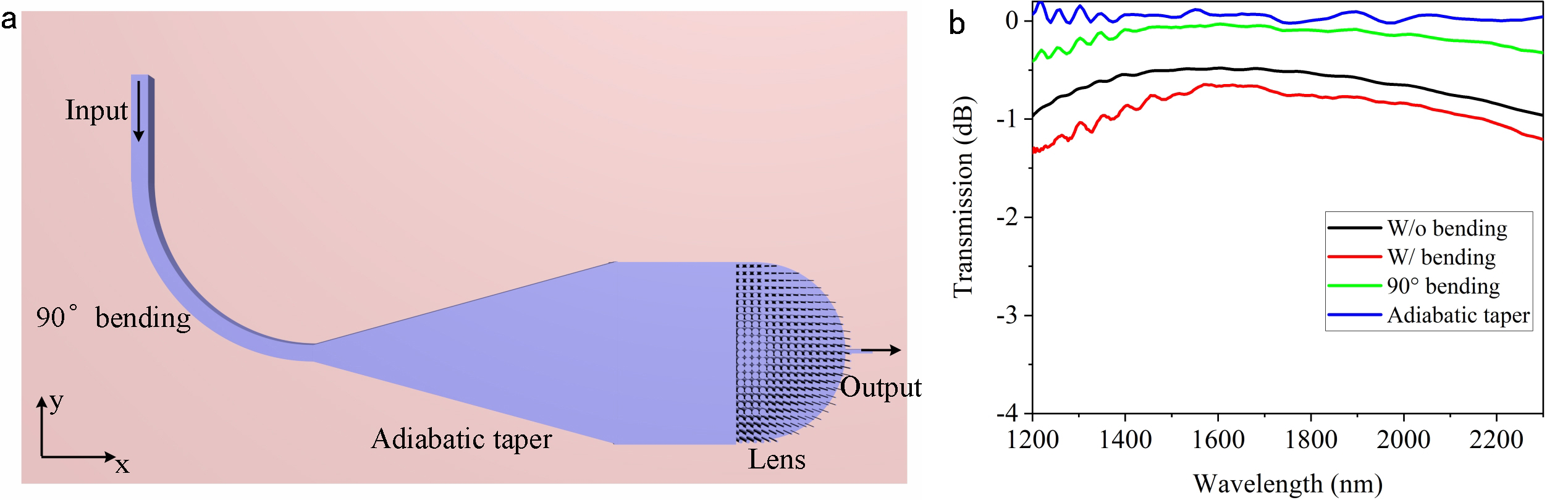


**Figure. S9:** Device characterization with 90° bending waveguide. (a) Schematic of the device with a 90° bending waveguide. (b) Simulated transmission spectra of the device with and without the 90° bending waveguide.

# References

[1]. J. M. Luque-González, R. Halir, J. G. Wangüemert-Pérez, J. de-Oliva-Rubio, J. H. Schmid, P. Cheben, Í. Molina-Fernández, A. Ortega-Moñux. An ultracompact GRIN‐lens‐based spot size converter using subwavelength grating metamaterials. Laser Photonics Rev. 2019; 13: 1900172.

[2]. L. H. Gabrielli, J. Cardenas, C. B. Poitras, M. Lipson. Silicon nanostructure cloak operating at optical frequencies. Nat. Photonics 2009; 3: 461-463.

[3]. R. Halir, P. J. Bock, P. Cheben, A. Ortega-Moñux, C. Alonso-Ramos, J. H. Schmid, J. Lapointe, D. X. Xu, J. G. Wangüemert-Pérez, Í. Molina-Fernández, S. Janz. Waveguide sub-wavelength structures: a review of principles and applications. Laser Photonics Rev. 2015; 9: 25-49.

[4]. J. M. Luque-González, A. Herrero-Bermello, A. Ortega- Moñux, Í. Molina-Fernández, A. V. Velasco, P. Cheben, J. H. Schmid, S. Wang, R. Halir. Tilted subwavelength gratings: controlling anisotropy in metamaterial nanophotonic waveguides. Opt. Lett. 2018; 43: 4691-4694.
